# Relationship satisfaction and interpartner agreement about acts of physical and psychological aggression: a multilevel analysis

**DOI:** 10.1186/s12888-017-1452-6

**Published:** 2017-08-15

**Authors:** José Luis Graña, María Luisa Cuenca, Natalia Redondo

**Affiliations:** Facultad de Psicología, Universidad Complutense, Campus de Somosaguas, 28223 Madrid, Spain

**Keywords:** Partner aggression, Agreement, Reliability, Relationship satisfaction, Dyadic data

## Abstract

**Background:**

To analyze, in a multilevel context, the impact of individual-level relationship satisfaction on couples’ mean reports of aggression and agreement about acts of physical and psychological aggression.

**Methods:**

We conducted a quota sampling method to recruit a community sample of 2.988 heterosexual adult couples from the Region of Madrid (Spain).

**Results:**

The percentages of intimate partner aggression considering the highest report of aggression in the couple were around 60% of psychological aggression and 15% of physical aggression. Couples that used aggressive tactics showed low to moderate levels of agreement about physical and psychological aggression. Multilevel models confirm that women’s relationship satisfaction had a significant influence on the level of agreement about acts of psychological aggression, but the same pattern of results was not observed for men. On the other hand, men and women’s relationship satisfaction had no significant influence on the level of agreement about physical aggression.

**Conclusions:**

Psychological aggression plays a more relevant role in women’s relationship satisfaction than physical aggression.

## Background

Research in the field of social sciences about certain phenomena of social interaction (i.e., love, conflict, aggression, satisfaction, or personal perception) highlights the importance of the interdependent nature of the variables in order to obtain an accurate estimate of such phenomena [1]. In this line, a methodological criterion that researchers and clinicians commonly use is to collect self-report data and evaluate the degree of interjudge agreement. However, self-report measures are subject to biases, and this criterion not only compromises the reliability of a measure, but also its validity [2].

This methodological aspect is important when estimating the prevalence of certain phenomena occurring in the private sphere of intimate relationships, as might be the case of partner aggression. This is especially relevant in clinical and forensic contexts and even in community settings where much of the research conducted with the Conflict Tactics Scale (CTS; [3, 4], the most widely used scale to measure incidence and prevalence of violence in intimate partner relations (for a review, see [5]).

The research in this area argues that it is quite apparent that both men and women use physically aggressive tactics during disagreements ([6] (p480)). Authors such as Capaldi and Langhinrichsen-Rohling (2012) considered this aspect “a critical dimension of intimate partner violence (IPV).” ([7] (p323)) Kelly and Johnson (2008) conceptualized this type of IPV as situational violence, a pattern of aggressive behavior that “is not based on relationship dynamics of coercion and control, is less severe, and mostly arises from conflicts and arguments between the partners.”([6] (p481)) From the point of view of gender theory, these authors argue that some types of IPV have greater gender symmetry (i.e., situational couple violence) than others (i.e., coercive controlling violence) ([6] (p487)) and, for this reason, some authors emphasize that “IPV is not a unitary phenomenon” ([8] (p187)).

This aspect has been systematically confirmed in the research performed, so that in “large-scale surveys research, using community or national samples, reports gender symmetry in the initiation and perpetration by men and women of IPV” ([6] (p481)). (For a review, see [9–11]).

Given that in representative and community samples of the general population the true prevalence rates of *physical aggression* are unknown [9–12] research conducted with the CTS [3, 4] has addressed the limitations raised by self-report measures, examining interpartner agreement about acts of aggression. However, agreement may not only vary depending on the method used to estimate it, but also on the characteristics of the study sample [13]. Consequently, agreement is also an important limitation in the estimation of this phenomenon, highlighting the existence of a number of biases that can influence men and women when reporting their own acts of aggression or those of the partner. Examples of such bias are: “social desirability, the perception that acts of aggression have fewer consequences in the partner than in oneself” ([14] (p13), [15, 16]) or the possibility that relationship satisfaction or dissatisfaction influences the concordance of partners’ reports [17].

The use of dyadic data is another way to deal with the limitations of self-report measures in the estimation of partner aggression, as they allow us to consider the higher aggression report of the dyad. This highest report has become known as the *maximal dyadic report* of intimate partner aggression [18, 19]. Using the dyadic report, one can capitalize on the interdependent nature of dyadic data [1].

O’Leary and Williams (2006) [18] examined a community sample of 453 married couples, finding that prevalences of aggressive acts based on the maximum dyadic report were higher than men and women’s individual perpetration and victimization reports. Furthermore, the results highlighted the potential disagreement about acts of aggression between the couple members. Other studies have examined, in couples in which at least one partner reported aggression, the influence of relationship satisfaction on couples’ mean reports of aggression and agreement [17]. The study results showed that, both for men and women, relationship satisfaction was an important bias that influenced the levels of agreement about physical and psychological aggression in couples experiencing relationship difficulties.

The variable relationship satisfaction has been analyzed in different contexts. In community samples, it has been inversely associated with psychological aggression [20–22] and, in couples seeking therapy for their relationship conflicts, psychological aggression is a significant predictor, not only of separation or divorce [23], but also of physical aggression [24]. On the other hand, for newlywed couples, transverse and longitudinal research has linked physical aggression with low levels of satisfaction [25–27]. Given the importance of the phenomenon of gender-based violence in Spanish society, this study aimed to determine the role played by the couple’s satisfaction with their relationship in partner violence and possible interventions derived from the results.

## Hypotheses

1. Given the similarity of US and Spanish cultures in the prevalence of intimate partner aggression [28] and that the percentages of male to female and female to male *physical aggression* was approximately 12% in representative community samples in US and Spain, it was predicted that, considering couples in which at least one partner reports (physical or psychological) aggression perpetrated either by the man or woman, the rates of female to male perpetrated physical aggression will be higher than rates of male to female, and the rates will be similar to those observed in community samples [10].
2. Given that the status of perpetrator/victim and gender are not sources of bias that influence reports of agreement about acts of partner aggression [15, 16, 18], we predict that these variables will not be a source of bias in community samples.
3. Given that low to moderate levels of agreement about acts of physical and psychological partner aggression have been observed in community samples [18], we expect to find low to moderate levels of interpartner agreement about psychological and physical aggression.
4. Given the importance of intimate relationships for satisfaction and happiness in Spain (CIS; Barometer June 2013), we expect that relationship satisfaction, both for men and women, has a significant impact on reporting agreement, people who are satisfied with their couple relationship will tend to report less presence of aggression--their own or their partner’s--whereas people who are dissatisfied with their relationship will tend to report a greater presence of acts of aggression.

## Method

### Participants

The participants of the study consisted of 2.988 adult heterosexual couples, aged between 18 and 80 years, from the region of Madrid. The inclusion criteria were being over 18 years of age and being in a heterosexual couple relationship either currently or in the past 12 months.

The majority (61.7%) of the participants were married; 29.5% were single with a partner but not cohabitating, 7.0% were common-law couples, and 1.8% were widowed, separated, or divorced and living with a partner. Men’s mean age was 40.44 years (*SD* = 14.00) and women’s mean age was 39.73 (*SD* = 13.93). The average relationship duration was 18.45 years (*SD=11.96*). Of the sample, 97% were Spanish, and 3% were of other nationalities. Concerning occupation, 43.1% were employees, 13.5% were civil servants, 11.4% were self-employed or autonomous workers, 8% were businessmen, 21.7% were unemployed, and 2.3% were students ([29] (p4)).

### Instruments and variables

*Sociodemographic Questionnaire*. Diverse items were included to assess participants’ characteristics in the following sociodemographic and personal variables: age, sex, civil status, nationality, professional activity, and current partner’s sex and age ([14] (p4), [30] (p2)).

*CTS-2. Revised Conflict Tactics Scale* [4]. We used the Spanish version of the CTS-2 by [31]. It is a self-report questionnaire with 39 duplicate items, that is, 39 questions as the perpetrator and 39 questions as the victim (78 items in total), on which participants rate the degree to which each member of the couple performs specific acts of physical, psychological, and sexual violence against the other partner, in addition to their use of justifications and negotiations to solve their conflicts.

The respondent of the CTS-2 scale should indicate how often he/she has carried out the acts mentioned in each item and how often his/her partner has carried them out. The response format ranges from 1 (*once in the past year)* to 6 *(more than 20 times in the past year*); 7 means *never in the past year but it used to occur before* and 0 means *it has never occurred.* For each item, participants indicate how frequently the incident has occurred in the past year. The main scores of the scale are:

*Prevalence*: these are dichotomic scores reflecting whether a participant reports the presence of a behavior defined in the scale in the past year. It is calculated by transforming responses 1-6 to 1, and responses 7 and 0 to 0. The item scores are not added, so the prevalence for each subscale will be 1 or 0 [4].

The CTS-2 scale shows good psychometric properties for the Spanish adult population [31]. Cronbach’s alphas on the total scale were .84 and .83, for perpetration and victimization, respectively. Furthermore, the alpha values for the remaining scales were: Negotiation (α = .76 and α = .75, for perpetration and victimization, respectively), Psychological Aggression (α = .72 and α = .73), Physical Aggression (α = .79 and α = .80), Sexual Aggression (α = .62 and α = .63), and Injuries (α = .75 and α = .69) ([14] (pp4,5), [29] (pp5,6), [30] (pp2,3)).

*QMI. Quality Marriage Index* [32]. The QMI consists of six items. On the first five items, respondents rate their degree of agreement with each statement (e.g., “My relationship with my partner makes me happy”) on a 7-point scale ranging from 1 (very strongly disagree) to 7 (very strongly agree). For the sixth question, respondents report how happy they perceive their relationship to be on a scale of 1 (unhappy) to 10 (perfectly happy). High scores indicate overall satisfaction with one’s marriage. Alpha coefficients for men and women’s reports were .91 and .90, respectively. Average inter-item correlations for men and women were .66, 95% CI [.55, .78] and .64, 95% CI [.54, .77], respectively.

### Procedure

The study used a quota sampling method to recruit a community sample of married or cohabitating couples from the Region of Madrid. In order to obtain the most representative sample possible of the active population of the diverse urban areas, the research assistants (RAs) were selected from 300 candidates from the Department of Clinical Psychology of the Complutense University of Madrid, who wished to obtain research credits, and every year, there were new RAs. We took as reference for the distribution of the RAs the population as a function of census of the Region of Madrid.

Each year, the research team organized an informative meeting with the RAs. The purpose of the meeting was to provide information about the characteristics of the study and the procedure to follow to recruit the study sample. A specific training was carried out with role-playing on how to approach the couples and ask for their voluntary participation so each one of the couple members would complete the questionnaire independently. We provided information on how to proceed if a victim of abuse was discovered, providing all the RAs with a resource guide for female victims of gender violence and a guideline of assistential resources for the aggressor members.

While conducting the present study, both the director and another member of the research team were available 6 h per week to carry out a follow-up of its evolution, which lasted 3 months per year.

The procedure was as follows: (a) each RA had to collect a quota of 8 couples from the assigned census area, 1/3 of whom could be acquaintances and the rest unknown, that had to be approached mainly by telephone calling and asking them if they wanted to participate in this study, and participants were told that they could give their consent by completing the questionnaire and sending it anonymously and independently of their couple to a PO Box; (b) the couples were selected taking into account the following age range:18-29, 30-50, and +50; (c) after obtaining the study quota, the RA had to give the code of each couple member to the director of the project (e.g., 1-a and 1-b up to 8-a and 8-b) and the phone number or email address of each couple, and (d) to confirm the veracity of the data, a random control of 10% of the participants of the study was performed.

The missing data were replaced through Expectation-Maximization (EM) procedure (SPSS version 21.0). The prevalence statistics reported in the present study are based on valid cases (i.e., missing data were not replaced prior to computing this statistic, and as no differences were obtained then, they were replaced with imputed values) ([14] (pp5,6), [29] (pp6-8), [30] (p3)).

### Data analysis

Analyses were completed using SPSS, version 19.0 and the HLM-7.0 program [33], a specific program to estimate multi-level models.

### Measures of agreement

In accordance with Marshall et al. (2011), [17] to examine agreement about acts of physical and psychological aggression in the couple, we calculated three concordance indices, such as the: Kappa κ [34], Yule’s *Y* coefficients and *d* prime (*d*´) [35]. These statistics have often been used in previous studies to assess this aspect (e.g. [17, 20, 36]).

Kappa and Yule’s *Y* coefficients are interpreted in a range of scores going from - 1 to +1, and the results are interpreted according to the following norms: <.0 = poor, .0-.2 = slight, .2-.4 = fair, .4-.6 = moderate, .6.-.8 = substantial, and .8-1.0 = perfect [37–39]. We also calculated the d´ statistic [40], an indicator used less frequently in the study of agreement (for an exception, see [41, 17]), but it allows examining the trend shown in the reports of aggression in the couple. To interpret this statistic, we used the norms for effect size of Cohen’s *d* (1988) [42] (i.e., .20 = small, .50 = medium, .80 = large).

### Correlates of agreement

To examine the impact of relationship satisfaction on reported agreement about physical and psychological aggression, we used multilevel modeling (HLM 7.0; [33]). According to the study conducted by Marshall et al., (2011), all the multilevel analyses were performed considering the couples who reported aggression (physical or psychological) ([17] (p368)).

In accordance with the objectives of the study, firstly, we created two parallel scales from the items that make up the scales of physical and psychological aggression of the CTS-2 in men and women, respectively. Conjointly, each parallel scale (a total of 4) is made up of half of the original scale’s items. To obtain the parallel scales for psychological and physical aggression an equivalent proportion of items were selected for each subscale considering the highest and lowest standard deviations. The assignment of items to each subscale was random, so each subscale has a similar variance and reliability. The results obtained will show the average acts of aggression and agreement in the couple and the subscale used will be multiplied by 2.

The total frequency scores of the CTS-2 were used to compare reports of aggression in men and women.

The Cronbach alpha coefficients for men and women on the physical aggression scale ranged from .65 to .67 and from .58 to .66, respectively. On the psychological aggression scale, the alpha coefficients ranged from .53 to .49 and from .56 to .56, respectively.

Secondly, we proposed the Level 1 and Level 2 equations resulting from the multilevel analysis. The equation of Level 1 was:

$$ {\mathrm{Y}}_{\mathrm{ij}}={\upbeta}_{0\mathrm{j}}+{\upbeta}_{1\mathrm{j}}(partner)+{r}_{ij}. $$

The terms of the equation are as follows:

- Y_ij:_ represents the report of perpetration of aggression (physical or psychological) informed by a man or a woman - *i* - in the couple - *j-* (1-2.988 couples).
- Β_0j_: represents the mean of a certain form of aggression (physical or psychological).
- *Partner*: variable coded as −0.5 for women and +0.5 for men, allows us to know the mean of couple aggression in men and women.
- β_1j_ (slope): represents an indicator that shows the direction of the agreement between the members of the couple.
- *r*_*ij*:_ represents the residual variance*.*

The equation of Level 2 was:

$$ {\upbeta}_{0\mathrm{j}}={\upgamma}_{00}+{\upgamma}_{01}\left(\mathrm{Man}\ \mathrm{QMI}\right)+{\upgamma}_{02}\left(\mathrm{Woman}\ \mathrm{QMI}\right)+{\upmu}_{0\mathrm{j}} $$

$$ {\upbeta}_{1\mathrm{j}}={\upgamma}_{10}+{\upgamma}_{11}\left(\mathrm{Man}\ \mathrm{QMI}\right)+{\upgamma}_{12}\left(\mathrm{Woman}\ \mathrm{QMI}\right)+{\upmu}_{\mathrm{ij}} $$

The terms of the equation are as follows:

- Β_0j:_ represents the mean report of aggression in the couple.
- Β_1j_: represents the report of agreement in the couple.
- γ_01_ and γ_02:_ represent the influence of each partner’s QMI score on couples’ mean aggression report.
- γ_11_ and γ_12:_ represent the influence of each partner’s QMI score on the agreement between partners’ aggression reports.

We used Cohen’s (1988) [42] guidelines for interpretation of effect size *d* .

## Results

### Descriptive statistics

In the past year, at least 1 partner reported male-perpetrated physical aggression in each of 424 couples (14.2%), and at least 1 partner reported female-perpetrated physical aggression in each of 458 couples (15.3%). Based on highest report given by either partner, these couples reported 1 to 109 acts of male-perpetrated physical aggression (*Mdn* = 2.0, *M* = 7.8, *SD* = 13.9), and 1 to 140 acts of female- perpetrated physical aggression (*Mdn* = 2.0, *M* = 7.5, *SD* = 15.1) in the past year. At least 1 partner reported male-perpetrated psychological aggression in each of 1081 couples (60.3%), and at least 1 partner reported female-perpetrated psychological aggression in each of 1903 couples (63.7%) in the past year. Based on highest report given by either partner in the past year, these couples reported 1 to 133 acts of male-perpetrated psychological aggression (*Mdn* = 8.0, *M* = 15.4, *SD* = 15.4), and 1 to 129 acts of female-perpetrated psychological aggression (*Mdn* = 8.0, *M* = 16.3, *SD* = 20.2) ([29] (pp9,10)).

### Partner agreement

As indicated in Table 1, 35% and 37% of couples agreed about the occurrence of male- or female-perpetrated physical aggression, respectively (85% and 86% of all couples), and 72% and 70% of couples agreed about the occurrence of male- and female-perpetrated psychological aggression (69% and 68% of all couples).

**Table 1:** Interpartner agreement about physical and psychological aggression perpetration

Perpetrator	Aggression	Aggressive couples	All couples	*K*	*Yules´ Y*	*d´*
		% Agreement	% Agreement			
Male	Physical	35.0	84.6	.24	.39	0.63
	Psychological	72.2	68.6	.35	.36	1.07
Female	Physical	36.8	85.6	.30	.45	0.72
	Psychological	70.1	67.7	.33	.34	0.97

Measures of *Kappa* and *Yule’s Y* indicate that agreement is low to moderate for male and female perpetrators of physical and psychological aggression. The measures of *d’* were similar for male and female perpetrators of physical aggression and higher for male perpetrators of psychological aggression.

### Multilevel modeling of agreement

“The residual terms for each Level 1 model were significant, indicating significant variation across couples to be explained by the Level 2 models” ([17] (p370)).

In physical aggression, the correlations between the mean reports of aggression and agreement in the couple were positive in men (*r* = .04) and women (*r* = .44). This result indicates that women reported the presence of more acts of physical aggression than their partners. In contrast, the correlations in psychological aggression were negative in men (*r* = −.01) and women (*r* = −.11), a result that indicates that women reported the presence of fewer acts of psychological aggression than their partners.

### Relationship satisfaction and physical aggression

In physical aggression, the reliability of the mean reports in the couple of aggression (β_0_) and agreement (β_1_) for men was .81 and .71, respectively. In the case of women, it was .11 and .10, respectively.

As indicated in the top portion of Table 2, the couples who informed of the presence of acts of physical aggression perpetrated by the man in the past year reported an average of 4 acts of physical aggression (β = 2.05 multiplied by 2 due to the use of parallel scales). Taking as reference the report of agreement, men reported having committed an average of 4 more acts of physical aggression than reported by their partners (β = 2.01 multiplied by 2 due to the use of parallel scales). This effect was statistically significant and was represented by a medium effect size (*d* = 0.32).

**Table 2:** Physical aggression. Level 2 models

	Male-perpetrator				Female-perpetrator			
Fixed effects	*β*	*SE*	*t*	*d*	*β*	*SE*	*t*	*d*
Couple mean								
Intercept	2.05	0.17	12.10***	.44	2.46	0.19	12.31***	.45
Man’s satisfaction	0.04	0.02	1.32	.05	−0.05	0.04	−1.24	.04
Woman’s satisfaction	−0.02	0.04	−0.57	.02	0.02	0.04	0.57	.02
Couple agreement								
Intercept	2.01	0.23	8.83***	.32	−1.56	0.59	−2.66***	.09
Man’s satisfaction	0.02	0.05	0.51	.02	−0.16	0.14	−1.12	.04
Woman’s satisfaction	−0.07	0.04	−0.67	.02	0.07	0.08	0.79	.03

*** p < .001

Couples who reported the presence of acts of physical aggression perpetrated by the woman in the past year informed of an average of 5 acts of physical aggression (β = 2.46 multiplied by 2 due to the use of parallel scales). Considering the report of agreement, women reported committing an average of 3 fewer acts of physical aggression than reported by their partners (β = −1.56 multiplied by 2 due to the use of parallel scales). This effect was represented by a small effect size (*d* = 0.09).

Neither partner’s relationship satisfaction was associated with couples’ mean or differential reports of male and female-perpetrated physical aggression

### Relationship satisfaction and psychological aggression

In psychological aggression, the reliability of the mean reports in the couple of aggression (β_0_) and of agreement (β_1_) for men was .39 and .36, respectively. In the case of women, it was .15 and .10, respectively.

As indicated in the top portion of Table 3, couples who reported the presence of psychological aggression perpetrated by the man in the past year, reported an average of 8 acts of psychological aggression (β = 3.96 multiplied by 2 due to the use of parallel scales). Taking as reference the report of agreement, men reported having committed an average of 3 more acts of psychological aggression than reported by their partners (β = 1.72 multiplied by 2 due to the use of parallel scales), as represented by a small effect size (*d* = 0.11).

**Table 3:** Psychological aggression. Level 2 models

	Male-perpetrator				Female-perpetrator			
Fixed effects	*β*	*SE*	*t*	*d*	*β*	*SE*	*t*	*d*
Couple mean								
Intercept	3.96	0.09	41.24***	1.50	4.79	0.09	48.91***	1.79
Man’s satisfaction	0.02	0.02	1.01	.04	0.01	0.02	0.46	.02
Woman’s satisfaction	−0.002	0.02	−0.11	.00	−0.02	0.02	−0.83	.03
Couple agreement								
Intercept	1.72	0.59	2.90***	.11	−2.96	0.58	−5.20***	.19
Man’s satisfaction	0.05	0.17	0.27	.00	0.01	0.12	0.10	.00
Woman’s satisfaction	−0.37	0.17	−2.16*	.08	0.02	0.04	2.65*	.09

* *p <* .05 *** *p <* .001

Women’s relationship satisfaction was significantly associated with couples’ agreement reports of psychological aggression as represented by a small effect size (*d* = 0.08). The direction of this effect indicates that women who were less satisfied with their relationship reported more acts of psychological aggression by their partners than their partners reported, and women who were more satisfied with their relationship reported fewer acts of psychological aggression than their partners reported. In the case of the men, satisfaction with the relationship was not significantly associated with reports of psychological aggression. The same was observed in couples that reported psychological aggression by the man and the woman, respectively.

Couples who reported the presence of psychological aggression perpetrated by the woman in the past year informed of an average of 10 acts of psychological aggression (β = 4.79 multiplied by 2 due to the use of parallel scales). Considering the report of agreement, women reported having committed an average of 6 fewer acts of psychological aggression than indicated by their partners (β = −2.96 multiplied by 2 due to the use of parallel scales). On average, partners reported significantly different levels of female-perpetrated psychological aggression, as represented by a small effect size (*d* = 0.19). Women’ lower relationship satisfaction was associated with agreement of reports, as represented by a small effect size (*d* = 0.09). The direction of this effect indicates that women who were more satisfied with their relationship reported committing fewer acts of psychological aggression than their partners reported, and women who were less satisfied reported perpetrating more acts of psychological aggression than their partners reported. In the case of the men, satisfaction with the relationship was not significantly associated with reports of psychological aggression. The same was observed in couples that reported psychological aggression by the man and the woman, respectively.

## Discussion

The present study analyzed psychological and physical aggression in heterosexual couples and examined, with multilevel models, the impact of individual-level relationship satisfaction on couples’ mean reports of aggression and agreement in a Spanish community sample.

In previous investigations, the high prevalence of psychological aggression in relationships has been confirmed [30, 18, 20, 43], results that are confirmed in the present study, as 60% of the couples reported the presence of acts of psychological aggression compared to 15% who reported the presence of physical assault (couples most frequently reported a “minor” severity level of aggression).

In the present study, we considered the information provided by each member of the couple about the presence of acts of physical and psychological aggression, so taking the couple as a unit of analysis offers a more accurate view of this phenomenon.

We found low to moderate levels of agreement on physical and psychological aggression. These results corroborate the evidence observed in previous research in community samples and extends our knowledge about this aspect from prior research conducted with the CTS-2 [44, 16–18].

Gender and victim/perpetrator status did not influence the direction of reports of physical and psychological aggression. However, we observed the same tendency as [17], in the sense that victim/perpetrator status was a predictor of the frequency of aggression and its increase. Specifically, we observed a tendency in men to report perpetrating more acts of physical and psychological aggression than reported by their partners. As for women, we observed a tendency to report perpetrating fewer acts of physical and psychological aggression than their partners reported. Moreover, regarding acts of physical aggression, this tendency was associated, both for men and women, with a similar average frequency of perpetration, even though the pattern of results observed was more reliable for men than for women. As for psychological aggression, this tendency was associated with a slight increase in the average frequency of acts of psychological aggression perpetrated by men, although the pattern of results observed was more reliable for men than for women.

Multilevel models confirmed that relationship satisfaction influenced reported agreement about psychological aggression, but only in the case of women. Furthermore, this pattern of results was consistent among couples who perpetrated psychological aggression. Indeed, psychological aggression is a concern in the case of women who are dissatisfied with their relationship because, in these cases, they tend to overestimate, not only the acts of psychological aggression perpetrated by their partners, but also the acts of psychological aggression perpetrated by themselves. However, it is more troubling in the case of women who are satisfied with their relationship because they tend to underestimate not only the acts of psychological aggression perpetrated by their partners but also the psychological aggression they themselves perpetrate.

These results can be explained considering that relationship satisfaction may have a different meaning for men and women and, therefore, a different impact when reporting one’s own acts of aggression or those of the partner, because, in the case of men, relationship satisfaction was not associated with agreement about physical and psychological aggression. In this sense, Marshall et al. (2011) [17] point out that men and women’s general schema of relationship satisfaction may be different and, in the case of women, general schema satisfaction may play a more important role. It may be that women with lower relationship satisfaction have no trouble accepting their involvement in certain acts of psychological aggression. In addition, tolerance has been considered to play a role in the couple’s satisfaction, so that, in the case of women, less satisfaction with the relationship may imply greater tolerance towards acts of psychological aggression. This aspect could influence conflict- resolution through psychological aggression [29]. Another explanation is possible, in line with *Cognitive Dissonance Theory* (Festinger, 1957), women who were less satisfied with their couple relationship used dissonance to justify the presence of these aggressive behaviors. Satisfied women used dissonance to ignore and justify the presence of these aggressive behaviors.

Previous research has considered other possible interpretations “such as the perception that acts of psychological aggression have less impact on one’s partner than on oneself, social desirability, certain motivations or attributions of responsibility and blame, or how partners remember relationship conflicts and how they resolve them, among other aspects” ([29] (p14)).

Perhaps men and women have no problems identifying either their own or their partner’s behaviors of psychological aggression. However, we have observed that, in the case of men, their level of satisfaction does not influence this type of aggression. A possible explanation of these data is that men may have developed a process of greater tolerance, acceptance or forgetfulness of certain acts of psychological aggression, which does not occur in the case of women, perhaps due to the greater sensitivity of this type of acts on women’s level of satisfaction (e.g., 50% of the couples had been involved in this relationship for 18.45 years). These results fits well into the clinical literature which has long suggested that women are better barometers of their relationship than men. [45].

However, the results obtained do not allow us to draw definite conclusions about the role of individual-level relationship satisfaction and physical aggression, as it had no significant effect on the mean reports of aggression and agreement. These results are consistent with the characteristics of the study sample, as the prevalence of physical aggression in community samples is low [9, 10, 12].

## Conclusions 

The present study examines the nature of certain variables such as satisfaction with the relationship with regard to aggression. One of the main conclusions of this study was to determine the relationship between satisfaction with the relationship and its influence on reports of psychological aggression in the couple. The results show that, in women, satisfaction with the relationship plays a very important role in the reports of the nature of psychological aggression. For women, being satisfied with the relationship involves underestimating acts of psychological aggression. This result has implications, and it would be advisable to contextualize women’s responses to questionnaires about aggression in the couple taking into account the reported level of satisfaction. Thus, if the level of satisfaction is very low, this is compatible with a higher estimation of partner-aggression and, in contrast, if the level of satisfaction is high, the real level of aggression may be underestimated. This relationship has not been found in men, and their reports of aggression appear to be independent of their level of satisfaction. If we extrapolate these community data to the clinical or forensic population, we would need a detailed assessment of both members of the couple to achieve a more objective and accurate picture of the relationship in those cases where aggression and violence is the reason for demanding couple therapy, separation and divorce suits, in custody cases, or in situations of gender violence. Therefore, we highlight the importance of considering certain methodological aspects such as collecting formation from both members of the couple, including other more objective behaviors than satisfaction with the relationship in order to examine the phenomenon of aggression in relationships in greater depth. Finally, we recommend the use of dyadic data, as they allow us to estimate the dyad’s higher report of aggression, together with an appropriate methodology that enables us to examine the influence of certain individual-level variables. This is a key aspect, because it permits us to obtain a more accurate estimate of the phenomenon of partner aggression.
